# Microfluidic Nanomaterial Synthesis and In Situ SAXS, WAXS, or SANS Characterization: Manipulation of Size Characteristics and Online Elucidation of Dynamic Structural Transitions

**DOI:** 10.3390/molecules27144602

**Published:** 2022-07-19

**Authors:** Anan Yaghmur, Islam Hamad

**Affiliations:** 1 Department of Pharmacy, Faculty of Health and Medical Sciences, University of Copenhagen, Universitetsparken 2, DK-2100 Copenhagen Ø, Denmark; 2 Department of Pharmacy, Faculty of Health Sciences, American University of Madaba, Madaba 11821, Jordan; i.hamad@aum.edu.jo

**Keywords:** drug delivery, dynamic structural transitions, hard nanocrystals, liposomes, microfluidics, nanoparticles, reaction times, SAXS, SANS, WAXS

## Abstract

With the ability to cross biological barriers, encapsulate and efficiently deliver drugs and nucleic acid therapeutics, and protect the loaded cargos from degradation, different soft polymer and lipid nanoparticles (including liposomes, cubosomes, and hexosomes) have received considerable interest in the last three decades as versatile platforms for drug delivery applications and for the design of vaccines. Hard nanocrystals (including gold nanoparticles and quantum dots) are also attractive for use in various biomedical applications. Here, microfluidics provides unique opportunities for the continuous synthesis of these hard and soft nanomaterials with controllable shapes and sizes, and their in situ characterization through manipulation of the flow conditions and coupling to synchrotron small-angle *X*-ray (SAXS), wide-angle scattering (WAXS), or neutron (SANS) scattering techniques, respectively. Two-dimensional (2D) and three-dimensional (3D) microfluidic devices are attractive not only for the continuous production of monodispersed nanomaterials, but also for improving our understanding of the involved nucleation and growth mechanisms during the formation of hard nanocrystals under confined geometry conditions. They allow further gaining insight into the involved dynamic structural transitions, mechanisms, and kinetics during the generation of self-assembled nanostructures (including drug nanocarriers) at different reaction times (ranging from fractions of seconds to minutes). This review provides an overview of recently developed 2D and 3D microfluidic platforms for the continuous production of nanomaterials, and their simultaneous use in in situ characterization investigations through coupling to nanostructural characterization techniques (e.g., SAXS, WAXS, and SANS).

## 1. Introduction

**Figure 1:**
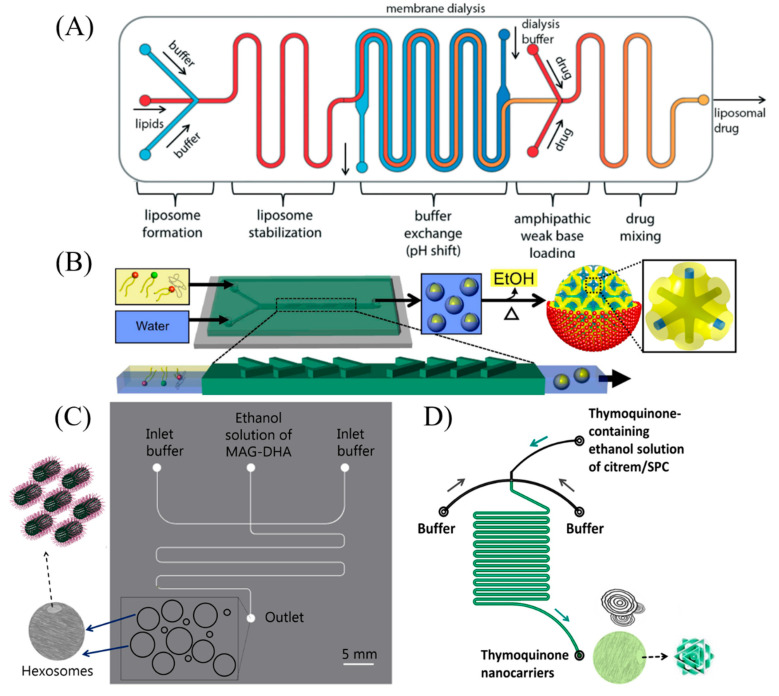
Schematic illustrations of continuous production of drug-free and drug-loaded lipid nanoparticles including liposomes, cubosomes, and hexosomes. (**A**) Multifunctional microfluidic device for rapid single-step microfluidic synthesis of monodispersed liposomes and their remote loading with therapeutics. Reprinted with permission from [27]. 2014, the Royal Society of Chemistry. (**B**) Staggered herringbone mixer for microfluidic synthesis of siRNA-loaded cubosomes. The continuous production includes additional chip-off step for evaporation of ethanol. Adapted with permission from [18]. 2018, the American Chemical Society. (**C**) Hydrodynamic flow-focusing (HFF) polyimide microfluidic device for microfluidic synthesis of Pluronic F127-stabilized hexosomes based on docosahexaenoic acid monoglyceride (MAG-DHA). Reprinted with permission from [19]. 2019, the Royal Society of Chemistry. (**D**) Continuous production of lamellar and non-lamellar liquid crystalline nano-self-assemblies (liposomes and cubosomes) for delivering the therapeutic agent thymoquinone by using a simple commercial microfluidic. Reprinted from [17]. 2021, MDPI.

Microfluidic platforms have emerged as attractive, powerful, and versatile tools for various biomedical and pharmaceutical applications, including nanomaterial synthesis, drug delivery, vaccine design, cell analysis, personalized medicine development, and diagnosis [1,2,3,4,5,6,7,8,9,10,11]. Among these, the continuous production of monodispersed nanomaterials (including soft lipid and polymer nanoparticles) and hard nanocrystals with controllable sizes and shapes is considered one of the frontline applications of microfluidics in recent years [1,5,7,12,13,14,15,16,17,18,19,20,21,22,23]. Schematic illustrations on the microfluidic synthesis of different nanoparticles attractive for use in the development of nanomedicines or functional food nanocarriers are presented in Figure 1. In addition to the microfluidic synthesis of monodispersed liposomes and their remote loading with therapeutics (Figure 1A), Figure 1B–D shows the employed microfluidic synthesis methods for the continuous production of cubosomes and other non-lamellar liquid crystalline nanoparticles (including hexosomes). These nanoparticles were produced through the use of a staggered herringbone mixer (Figure 1B) and hydrodynamic flow-focusing (HFF) microfluidic devices (Figure 1C,D), respectively. For further detailed information on these microfluidic methods for nanomaterial synthesis and recent advances in this research area, the interested reader is directed to the following recent review articles [5,20,21,24,25,26]. 

In addition to the high reproducibility and possible implantation of automation steps, microfluidics offers unique opportunities for online chemical and biological analyses, rheological characterization, and structural screening and analysis [5,8,9,21,28,29,30,31,32,33,34,35,36,37]. These opportunities include short measurement times, a high precision of liquid modulation and control of timing and flow parameters, and use of minimal input sample volumes (particularly important for minimizing the consumption of expensive materials) [4,7,12,16,31,32,38]. Here, 2D and 3D microfluidic platforms are attractive for real-time tracking analysis of nucleation and monitoring of growth mechanisms of hard metal nanocrystals [39,40,41,42,43], in situ (online), high-throughput protein structure screening and analysis during crystallization processes and under various conditions [44,45], real-time monitoring of structural dynamic events during the generation of nano-self-assemblies [5,28,30,31,46,47], and online self-assembly behavior of amphiphiles or macromolecules under flow conditions [44,46,48,49,50,51]. Further, the use is extended to other research areas, including in situ characterization studies for monitoring the dynamic structural events during the digestion of drug formulations [52], the exposure of charged nano-self assemblies to divalent ions [53,54], the spinning process of artificial fibers [55], the generation of single domain supercrystals [56], the precipitation of crystals in moving droplets [57], and the nanoparticle agglomeration [58]. 

There is a growing interest in coupling SAXS (or SANS) to light sources, stopped-flow devices, pressure cells, and optical tweezers, among others, for online structural characterization investigations [59,60,61,62,63,64,65,66,67,68,69]. However, in this review, we exclusively focus on in situ characterization of nano-self-assemblies under flow conditions through coupling of 2D or 3D microfluidic platforms to nanostructural characterization scattering techniques (namely synchrotron SAXS, WAXS, and SANS). Recent advancements in the fabrication of specialized microfluidic platforms with reduced attenuation and background scattering compatible to these techniques are discussed. Limitations, challenges, and opportunities in this research area are also highlighted. Different recent examples on in situ characterization studies (SAXS/WAXS-on-chip and microfluidic-SANS) are presented. The current number of such studies is limited, but it is expected to rapidly grow in the future. 

## 2. Two-Dimensional and Three-Dimensional Specialized and Compatible Microfluidic Chips for In Situ Characterization Studies

Coupling microfluidics to SAXS (or SANS) provides unique opportunities for investigating self-assemblies under confined and continuous flow conditions. In this section, we describe the main microfluidic characteristic features of 2D and 3D *X*-ray- and neutron-compatible microfluidic platforms designed for online (in situ) investigations on nanomaterials during their continuous productions or for real-time monitoring of phase behavior of surfactant solutions under flow conditions. Table 1 presents different examples from the literature on such online investigations and briefly describes the reported advantages of the employed microfluidic platforms. However, certain common microfluidic advantages (including simplicity, low cost, ease and rapid fabrication, reproducibility, flexibility, adaptability, and minimization of material consumption) are not mentioned in the table. 

The *X*-ray-compatible microfluidic platforms typically have different main characteristic features and advantages including: (i) *X*-ray transparency and compatibility; (ii) reduction of attenuation and background scattering and achievement of a good signal-to-noise ratio; (iii) reproducibility and negligible platform-to-platform variations; (iv) prevention the adsorption of lipids, proteins, and other compounds onto the interfaces of the microfluidic walls that may cause clogging and reduce the quality of measurements; (v) elimination of potential radiation damage; (vi) compatibility to organic solvents; (vii) ease of cleaning between measurements; and (vii) durability [28,42,43,49,57,70,71,72,73]. 

To guarantee successful SAXS analysis, it is important when employing HFF devices or similar microfluidic platforms to ensure that the channel width, through which the *X*-ray travels, is bigger than that of the *X*-ray beam for preventing parasitic scattering from the microfluidic channel walls [31]. Here, SAXS measurements at different positions along the channel should be conducted under flow conditions by maintaining the *X*-ray beam confined between the channel walls. The use of such devices with relatively bigger channel widths can compromise the microfluidic efficacy in controlling the nanoparticle size characteristics [31]. 

For online SANS investigations, it is important to use microfluidic chips with a low neutron absorption and a low neutron activation [37]. For further information, the interested reader is directed to the recent review of Cabral and co-workers [37]. It presents different microfluidic requirements (including some commonly shared with *X*-ray-compatible chips) [37]. 

For special and certain applications, we may need to consider additional microfluidic characteristic features for online SAXS (or SANS) studies [37].

**Table 1:** Examples of *X*-ray- and neutron-compatible microfluidics and their reported uses in online structural investigations.

Microfluidics	Features, Advantages, and Reported Main Drawbacks	Characterization and Phase Mapping	Ref.
2D HFF platform based on thiol-ene	Suitability for *X*-ray studies; lipid adsorption prevention; and disposability. Main drawback: low efficacy in controlling the nanoparticle size characteristics.	➢ Effect of Ca^2+^ ions on negatively charged cubosomes: synchrotron SAXS study. ➢ Continuous production and characterization of MLVs: synchrotron SAXS study. ➢ Mixing nanoparticles and micellar solutions: production of lamellar and non-lamellar liquid crystalline nanoparticles: synchrotron SAXS study.	[53] [31] [30]
Different polyimide- based chips	Good resistance to *X*-ray; suitability for SAXS-scanning studies: investigation of orientation and structural features of self-assemblies; thermal stability; and compatibility to organic solvents.	➢ Provision of in situ structural information on different soft matters (including orientation aspects). ➢ Behavior of lamellar and hexagonal phase under flow conditions.	[34] [74]
Cyclic olefin copolymer (COC) devices	Prevention of leakage (fabrication from COCs only, no need for gluing between interfaces); suitability for *X*-ray studies; and high *X*-ray transmission and radiation resistance. Main drawbacks: incompatibility with tetrahydrofuran and instability at relatively high temperatures [49].	➢ Monitoring early formation stage of well-ordered structures from self-organized intermediate filament proteins.	[73]
Laser lithography (LL) chips	High transparency and low *X*-ray background scattering; and suitability for *X*-ray studies.	➢ Characterization of phospholipid nanodispersion as a proof of concept.	[72]
3D polyimide chips	A more efficient and uniform mixing as compared to 2D polyimide chips; a combination of suitability for *X*-ray studies and compatibility to organic solvents with 3D focusing. The employed laser micromachining procedure is also reliable.	➢ In situ SAXS-on-chip investigations: mapping phase transitions within millisecond time scales under flow conditions.	[49]
Platform based on thiol-ene	Pressure and temperature resistance; capability to handle viscous fluids; suitability for *X*-ray studies. Interesting features: SAXS set-up allows controlling the temperature and conducting SAXS experiments at relatively high temperature.	➢ In situ SAXS-on-chip investigations on the structure and orientation of lamellar phases and MLVs based on surfactant solution. The online experiments are conducted at 70 °C.	[71]
A custom-built crown glass contraction– expansion device	Suitability for SANS studies; enabling tubular flow in continuous and oscillatory modes, relevance to industrial continuous and tubular flow processes.	➢ In situ SANS-on-chip investigations on transformation of lamellar phase to MLVs: structural elucidation and alignment behavior of a flow-responsive surfactant solution.	[51]
3D Kapton- based flow-focusing device	Compatibility to organic solvents; suitability for SAXS studies; a high spatial and temporal resolution; and rapid and efficient mixing of solvents.	➢ In situ SAXS-on-chip investigations on early clustering behavior of gold nanoparticles under flow conditions.	[43]
Droplet-based device	Suitability for *X*-ray studies; a high-throughput analysis; automatic screening of variable crystallization conditions.	➢ In situ SAXS-on-chip investigations on protein interactions and crystallization from solution.	[45]
Silicon/glass chips	High cost; suitability for SAXS and SANS studies; compatibility to organic solvents; and a time-consuming fabrication.	➢ In situ SAXS-on-chip investigations on lipid nanocapsules.	[47]
Epoxy-based chips	Good conditions for SAXS studies; *X*-ray and optical transparency; pressure resistance up to 2.9 bar; chemical resistance to certain solvents. Main drawbacks: incompatibility to some organic solvents, including tetrahydrofuran.	➢ SAXS-on-chip tests in absence of any sample.	[70]
Thiol-ene- epoxy (OSTE+) droplet devices	Suitability for SAXS/WAXS studies; *X*-ray transparency; *X*-ray signal quality of OSTE+ material as compared to typically used polyimide (Kapton).	➢ SAXS-on-chip investigations on gold nanoparticles. ➢ In situ characterization of cerium oxalate.	[57]

## 3. Continuous Production and In Situ Characterization of Nano-Self-Assemblies Attractive for Drug or Functional Food Delivery, or Vaccine Development 

**Figure 2:**
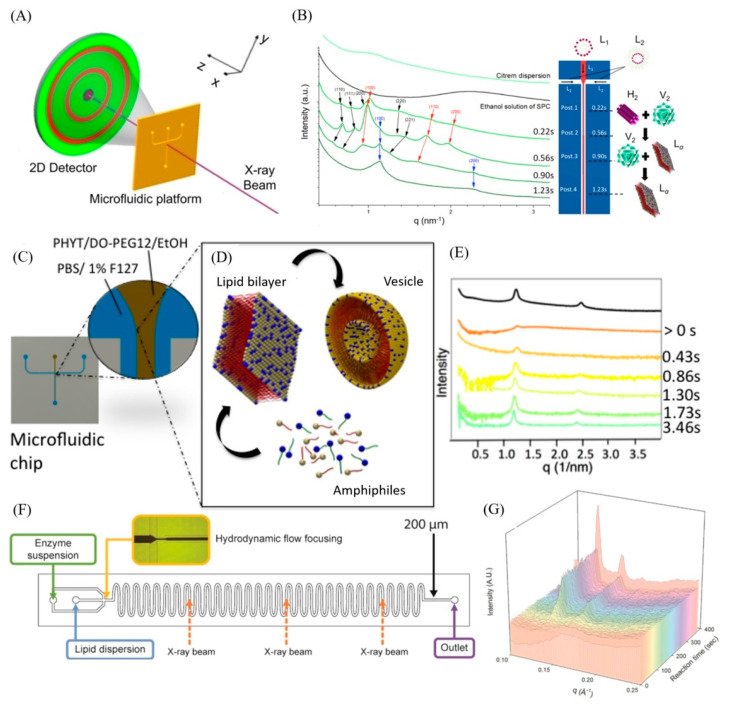
(**A**) Schematic illustration of an experimental set-up of coupling *X*-ray-compatible microfluidic platform to synchrotron SAXS. Reprinted with permission [5]. 2021, Elsevier. (**B**) SAXS patterns at different reaction times (different corresponding positions along the center channel of thiol-ene-based hydrodynamic flow-focusing (HFF) chip coupled to synchrotron SAXS. It indicates nonlamellar–lamellar phase transitions on exposure of citrem nanoparticles to an ethanol solution of soybean phospholipid. Adapted with permission from [30]. 2017, the Royal Society of Chemistry. (**C**) Continuous production of multi-lamellar vesicles (MLVs) through use of thiol-ene-based HFF chip. (**D**) Schematic illustration of the formation mechanism of vesicles in the HFF microfluidic chip. (**E**) In situ synchrotron SAXS characterization: SAXS patterns at different corresponding reaction times for SAXS measurements conducted at different positions along the center channel of the HFF chip (different corresponding reaction times) and compared with the black SAXS pattern of already prepared chip-off samples. Panels (**C**–**E**) reprinted with permission from [31]. 2017, the American Chemical Society. (**F**) In situ SAXS characterization of nano-self-assemblies generated during digestion of lipid formulations by coupling synchrotron SAXS to serpentine HFF microfluidic chip. SAXS measurements were conducted at different positions along the microchannel. (**G**) Digestion of lipid formulation containing phytantriol, leading to the evolvement of inverse cubic *Pn3m* phase after an elapsed time of about 90 s. Panels (**F**,**G**) reprinted with permission from [52]. 2019, the Royal Society of Chemistry.

**Figure 3:**
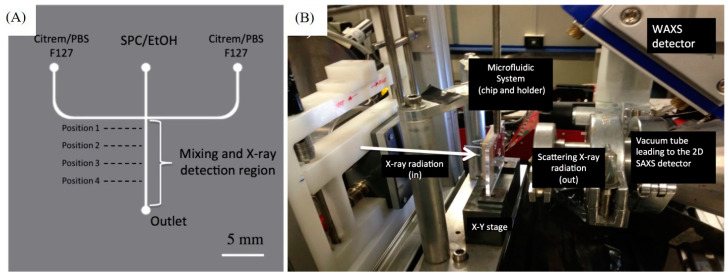
(**A**) Schematic illustration of an *X*-ray-compatible HFF microfluidic device for in situ SAXS characterization of lipid nanoparticles at 4 different positions along the microchannel. (**B**) Synchrotron SAXS set-up employed by coupling SAXS to an *X*-ray-compatible HFF microfluidic device for in situ characterization studies. The figure is reprinted with permission from [30]. 2017, the Royal Society of Chemistry.

This section presents different examples on coupling microfluidics with SAXS (or SANS) for the online characterization of nano-self-assemblies. Here, it is worth noting that the in situ characterization of nano-self-assemblies during their microfluidic synthesis, or on exposure of already off-chip-prepared ones to an external trigger (such as divalent ions), or an environmental change at different measurement time points (reaction times) and under confined microfluidic geometries, is still in its infancy. The number of articles on such online characterization investigations is still very modest. Clearly, there is need to expand the research efforts in this direction for gaining further insight into the dynamic structural events occurring during the continuous production of lipidic or polymeric nanoparticles that are attractive for use in various technological applications, including the development of drug (or functional food) nanocarriers, vaccines, and nanoreactors for chemical and enzymatic reactions. These studies will improve our understanding of the involved dynamic structural pathways and formation kinetics during the microfluidic nanoparticle synthesis process. They will also provide further insight into the behavior of already off-chip-prepared lipid formulations (including emulsions, liposomes, and solid lipid nanoparticles) during their digestion, interactions with biologically relevant media (such as human plasma), or on exposure to an external trigger. In this section, different studies on in situ characterization of nano-self-assemblies during their continuous production through use of microfluidic platforms are presented. Figure 2 shows a few different examples on such in situ nanostructural investigations through coupling of *X*-ray-compatible microfluidic platforms to synchrotron SAXS. Figure 3 presents an illustration of an employed *X*-ray-compatible HFF microfluidic chip with different probed SAXS measurement positions along the microchannel, and the employed experimental synchrotron SAXS set-up, respectively. 

As noted above, coupling synchrotron SAXS to *X*-ray-compatible microfluidic platforms, as illustrated in Figure 2A, provides a powerful in situ characterization tool for studying the early dynamic events of the microfluidic nanoparticle synthesis process. It is attractive for use for gaining insight into the dynamic phase behavior by mapping structural alterations and transitions and detecting in real-time the involved intermediate phases. For example, Khaliqi et al. [30] reported on coupling of synchrotron SAXS with an *X*-ray-compatible microfluidic device for online characterization of the early dynamic structural events, occurring on mixing an already chip-off-prepared citrem nanodispersion with a micellar solution of soybean phospholipid (Figure 2B). Such interfacing of a thiol-ene-based hydrodynamic flow-focusing (HFF) chip with synchrotron SAXS allowed, at different reaction (mixing) times (different corresponding positions along the microchannel), for in situ monitoring of fast nonlamellar–lamellar transitions within fractions of seconds (Figure 2B). These results demonstrated the rapid lipid exchange among citrem nanoparticles and micelles. Through varying lipid composition and ethanol concentration, and precise control of the experimental flow parameters, this microfluidic method is attractive for generation of lamellar (vesicles) and non-lamellar nano-self-assemblies (such as cubosomes and hexosomes) by rapidly mixing micelles with citrem nanoparticles prepared chip-off. The latter sample was prepared by employing a low-energy emulsification method (vortexing citrem in excess buffer). The same HFF chip was also coupled with synchrotron SAXS by Ghazal et al. [31] for online characterization of multi-lamellar vesicles (MLVs) during their microfluidic synthesis process at a constant flow rate ratio (FRR) of 17.2, which is the ratio of the sheath streams to the center stream, and total volumetric flow rates (TFRs) of 5 or 10 μL/min (Figure 2C). It was reported on rapid generation of MLVs on exposure of an ethanol solution containing a binary lipid mixture to excess buffer [31]. Herein, the vesicle microfluidic synthesis process involves most likely the pathways suggested by Lasic [75] and Jahn et al. [14,15]. These include the generation of dynamic intermediate flat and disk-shaped nanoobjects (bilayered lipid (or phospholipid) fragments (BPFs)) and their self-closure to vesicles (Figure 2D). It was proposed that the growth of these intermediate nanoobjects and their self-closure to vesicles are attributed to the self-assembly of the lipids with simultaneous diffusion out of the organic solvent (ethanol) molecules into the continuous aqueous medium. This is associated with mutual lipid and water diffusion with simultaneous environmental changes in the composition of the continuous medium on exposure of the organic solution of the lipids to excess buffer [15,31]. Further, it is worth considering the plausibility of the formation of an asymmetric curved state bilayer with non-zero spontaneous curvature (C_0_ ≠ 0) due to an uneven distribution of the embedded lipid molecules at the lipid–water interfacial film, leading eventually to the self-closure to vesicles [31,76,77,78]. The fast generation of the continuously produced vesicles is clearly seen in Figure 2E. They start to be evolved at approximately 0.43 s and their structures are fully developed within around 1–2 s.

In a recent study, Boyd and co-workers [52] reported on online characterization of lipid formulations during their digestion through coupling a simple T-junction microcapillary system, HFF microfluidic chip (Figure 2F), or pH-stat apparatus to synchrotron SAXS. They reported on the suitability of both microfluidic devices for the in situ SAXS characterization investigations of lipid nanoparticulate formulations. Figure 2G shows the SAXS patterns at different reaction times, indicating the generation of an inverse cubic *Pn3m* phase on digesting a lipid formulation containing phytantriol [52].

**Figure 4:**
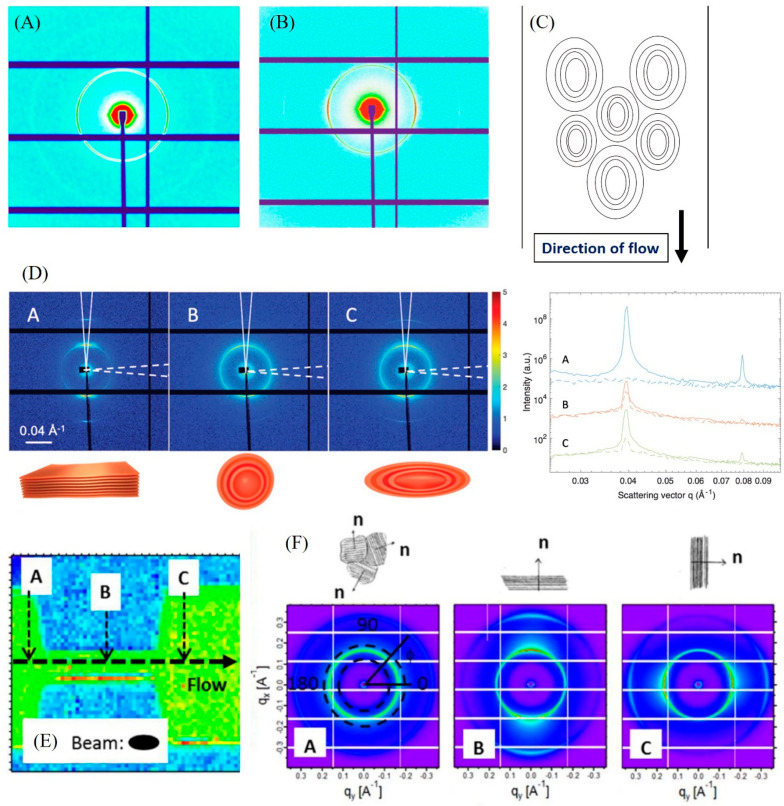
(**A**) 2D isotropic SAXS pattern during the formation of MLVs at TFR of 5 μL/min. (**B**) A light alignment of the MLVs (slightly distorted) at relatively high TFR (>15 μL/min). Slightly anisotropic SAXS pattern, indicating a slight deformation of the continuously produced MLVs as illustrated in (**C**). Panels (**A**–**C**) reprinted with permission from [31]. 2017, the American Chemical Society. (**D**) Left: 2D SAXS patterns of lamellar structures in three areas (A–C) with different orientations: an aligned lamellae structure, MLVs, and stretched MLVs in the flow direction. Right: Corresponding radial SAXS profiles in the three areas (A–C). Adapted with permission from [74]. 2021, Wiley. (**E**) SAXS measurements at the positions A–C along the centerline of the microchannel. (**F**) 2D SAXS patterns at three measurement positions (A–C): an isotropic pattern at position A; whereas a strong anisotropy was detected at positions B and C, indicating bilayers alignment upon entering the microfluidic constriction and upon exiting, respectively. Panels (**E**,**F**) reprinted with permission from [71]. 2016, the American Chemical Society.

It is worth also noting that the flow strains and the employed shear stresses in microfluidics may lead to the alignment of vesicles and affect the orientation of lamellar and hexagonal phases under shear flow, resulting in the appearance of anisotropic SAXS (or SANS) patterns [31,71,74,79]. For instance, a slight alignment was detected during the microfluidic synthesis of MLVs on increasing TFR from 5 to 15 μL/min (Figure 4A,B). A shear-induced deformation of MLVs was also detected under shear flow during their formation from a lamellar phase [79]. Through a microfluidic SAXS scanning study, Liebi and co-workers [74] reported on the flow-induced transformation of the lamellar (L_α_) phase to an aligned lamellae region via MLVs to an extended lamellae region by increasing the shear rates (Figure 4D). A significant effect of the microfluidic constriction, leading to an orientation of the lamellar phase under flow, was also reported (Figure 4E,F) in another study [71]. 

## 4. In Situ Phase Behavior and Structural Dynamics Investigations of Amphiphilic Polymers and Lipids 

In addition to the aforementioned reports on the in situ characterization of soft lipid (mainly vesicles) and polymer-based nano-self-assemblies under flow conditions, it is worth presenting different studies on coupling synchrotron SAXS (or SANS) with microfluidics for investigating the phase behavior of amphiphiles (including amphiphilic lipids and polymers). 

There is a growing interest in designing different microfluidic platforms for accurate and rapid mapping of phases of surfactant solutions [48,50,80]. For instance, a rapid phase-mapping method based on a microfluidic device with a stepped temperature profile was recently reported, and the identified phases were validated through the use of optical microscopy and SANS analyses [80]. In this section, we only focus on investigations conducted by coupling microfluidics to synchrotron SAXS or SANS. Among these online investigations, we mention the work of Kenis and co-workers [48] on on-chip formation and in situ SAXS characterization of lyotropic non-lamellar liquid crystalline phases through the use of four different microfluidic platforms with active-mixing capabilities and suitability for *X*-ray experiments [48]. This microfluidic method led to a significant reduction in materials used in lyotropic liquid crystalline preparations, and the obtained phases (including lamellar and inverse bicontinuous cubic phases) and their structural features agreed well with those produced chip-off [48]. In another study, it was reported on online scanning SAXS of lamellar (L_α_) and normal hexagonal (H_1_) phases in microfluidic channels [74]. It was found that these phases are aligned under flow, but the flow-induced changes are not identical. Increasing the shear rate was associated with the observation of a perpendicularly oriented H_1_ phase to the flow, and the presence of flow-induced orientation in the flow direction at relatively high shear rates. However, the flow-induced alignments and involved transitions were different for the L_α_ phase as discussed above (see Figure 4D). Other published examples on flow-induced orientations of liquid crystals (including lamellar and nematic phases) and cylindrical micelles are presented in the review of B. Silva [28] and the report of Trebbin et al. [81], respectively. 

There are also different recent studies on combining SAXS with 2D or 3D microfluidic platforms for investigating phase transitions in solutions based on amphiphilic co-polymers [49,50,82]. Among these studies, we mention the use of a 3D Kapton microfluidic device in combination with SAXS for online investigations of self-assembly of amphiphilic copolymers under flow conditions [49]. In another study, it was reported on fast self-assembly of an amphiphilic copolymer in microfluidics, leading to the formation of micelles and an FCC liquid crystalline phase [50]. 

For online SANS investigations, different microfluidic platforms were suggested for phase mapping of surfactant solutions [9,37,51,83,84,85]. For instance, a transformation of the lamellar liquid–crystalline phase to MLVs was recently investigated by employing microfluidic-SANS [51]. Further information on compatible microfluidics with SANS and recent online structural investigations is presented in the recent review of Lopez et al. [37]. 

## 5. In Situ Structural Dynamics and Kinetic Formation Investigations of Hard Solid Nanoparticles

**Figure 5:**
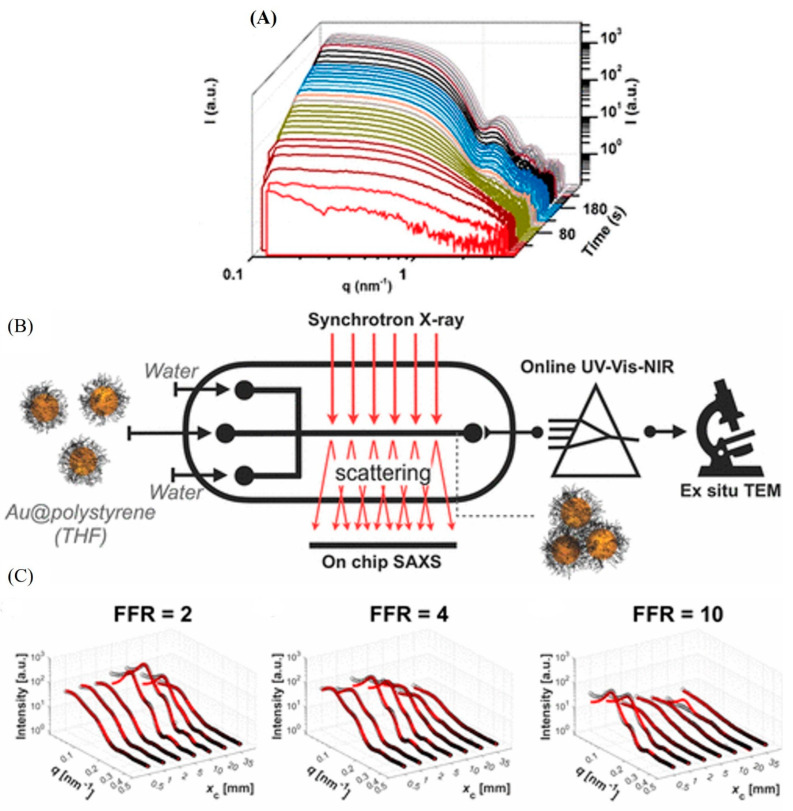
(**A**) SAXS patterns at different time points during the formation of gold nanoparticles in a stopped-flow microfluidic platform. (**B**) On-chip SAXS experiments and real-time UV–vis–NIR measurements for online studies of the self-assembly of gold nanoparticles and their clustering behavior upon mixing with water by using 3D flow-focusing microfluidic reactor. (**C**) On-chip characterization of nanoparticle clustering behavior. At 3 flow rate ratios (FFRs) of 2, 4, and 10, SAXS patterns are recorded at different downstream microfluidic channel positions. (**B**) reprinted with permission from [86]. 2021, the American Chemical Society. Panels (**B**,**C**) reprinted with permission from [43]. 2019, (the American Chemical Society.

In addition to the aforementioned online investigations, it is worth mentioning the growing interest in coupling SAXS (or SANS) with suitable microfluidic devices for the in situ characterization of hard solid nanoparticles, including nanocrystals, silk fibers, and millimeter-sized supercrystals [41,42,43,55,56,57,86]. For instance, Chen et al. reported through in situ UV–vis, and time-resolved microfocus SAXS experiments on the formation process of monodispersed gold nanoparticles with different sizes in a stopped-flow microfluidics (Figure 5A) [86]. They found that the nucleation and growth process are controlled by the binding affinity of the investigated ligands to the gold nanoparticles [86]. In another recent study, a 3D flow-focusing microfluidic reactor was combined with SAXS and UV–vis–NIR spectroscopy for the online characterization of gold nanoparticles coated with polystyrene. At an early stage, it was possible using this experimental set-up to gain important information on the collapse of the polymer shell and the clustering behavior of the nanoparticles (Figure 5B,C). A similar approach was also followed in the work of Herbst et al. [41]. They reported on coupling microfluidics to SAXS/WAXS and UV–vis spectroscopy for gaining insight into the nucleation and growth kinetics of ZnO nanoparticles. They introduced a microfluidic method for preparing monodispersed ZnO nanoparticles and discussed the involved intermediate states during their microfluidic synthesis. 

Another use of microfluidic-SAXS is in studies of the early-stage agglomeration of nanoparticles and their interactions on varying the composition or properties of the surrounding aqueous media of the investigated suspensions [58]. 

In addition to SAXS and SANS, coupling microfluidics to *X*-ray absorption microscopy (XAS) is another option with unique opportunities for the in situ monitoring of reactions and crystallization processes [87,88,89]. For instance, deMello and co-workers reported on the integration of a droplet-based microfluidic platform with XAS for in situ monitoring of calcium carbonate precipitation [87].

## 6. Conclusions

Microfluidics has various advantages (including versatility, small sample volumes, scale-up ease, and precise control over fluid conditions). It offers, therefore, unique opportunities for various technological applications and opens up new possibilities for uses in analytical and material science research fields. Among others, microfluidic platforms are powerful and attractive tools for the continuous production of nanomaterials with controllable sizes (including drug nanocarriers and solid crystalline and amorphous nanoobjects). 

Thanks to recent advances in the microfluidic research area, there is a growing interest in the use of 2D and 3D microfluidics in numerous online characterization investigations of soft and hard nanoparticles. Among different state-of-the-art tools, we present here recent advances on coupling SAXS (or SANS) to compatible microfluidics for gaining insight into the involved structural pathways and formation kinetics of soft nanoparticles and solid nanoobjects. Such in situ SAXS (or SANS)-on-chip studies provide also important information on the behavior of nanomaterials under confined geometries and flow conditions.

Despite the attractiveness of microfluidic devices and the expected significant increase in their uses in in situ SAXS (or SANS)-on-chip research investigations in the next 10 years, the design and use of suitable microfluidic platforms for such studies pose a major challenge. In this research area, these studies generally require experts with highly specialized skills, particularly when using microfluidics with complex structures, or when the operation and control of flow conditions involve multiple steps. In addition, it is worth noting the reported microfluidic platforms for online SAXS (or SANS) studies in the literature are typically custom-built or home-made systems, and accessible to relatively few research groups. However, different synchrotron SAXS and SANS facilities recently focus on the engagement of a larger community of researchers through the provision of access to *X*-ray- and neutron-compatible microfluidic platforms for performing time-resolved experiments during the beamtimes.

We should take into account that this area is multidisciplinary, and, therefore, further research progress is expected through the integration and collaboration of scientists with different backgrounds. It is also worth noting that the potential uses of SAXS (or SANS)-on-chip for online investigations are mainly explored in academic studies, whereas the engagement of industry is very limited. Here, more industry engagement and further industry–academia collaborations will contribute to this research area.
